# The social role of defective viral genomes in chronic viral infections: a commentary on Leeks et al. 2023

**DOI:** 10.1111/jeb.14244

**Published:** 2023-11-17

**Authors:** Lele Zhao, Katrina A. Lythgoe

**Affiliations:** ^1^ Big Data Institute, Li Ka Shing Centre for Health Information and Discovery, Nuffield Department of Medicine University of Oxford Oxford UK; ^2^ Pandemic Sciences Institute, Nuffield Department for Medicine University of Oxford Oxford UK; ^3^ Department of Biology University of Oxford Oxford UK

In their article, Asher Leeks and colleagues (2023) set out a series of important questions aimed at linking empirical virology with social evolution theory to help stimulate the growth of the field of ‘sociovirology’. The hope is that by re‐evaluating viral interactions in the context of social evolution, a new understanding of viral biology will emerge that will not only improve understanding of fundamental biology and clinical manifestations but could lead to new and innovative ways to control infectious pathogens. Here, we use the ideas and concepts presented by Leeks and colleagues (2023) as a springboard to focus on social interactions in three human chronic viral infections of immense public health importance, HIV‐1, hepatitis B virus (HBV) and hepatitis C virus (HCV).

If untreated, HIV‐1, HBV and HCV can lead to long‐term chronic infections, characterized by sustained high viral loads. They are also among the leading causes of death in low‐ and middle‐income countries and result in huge economic losses worldwide. A great deal of effort has been devoted to the basic pathology and clinical research of these viruses. Using the latest generation of direct‐acting antivirals, HCV is eradicated in more than 95% of treated infected individuals (Feld et al., 2015; Forns et al., 2017; Götte & Feld, 2016), and the successful treatment of HIV‐1 and HBV infections results in long‐term viral suppression. However, HIV‐1 and HBV are rarely cured, meaning individuals typically have to take long‐term medication to prevent viral rebound. And for both HIV‐1 and HCV, drug resistance is becoming increasingly prevalent, which is a real concern for future public health (Gupta et al., 2012; Howe et al., 2022).

In this commentary, we take a closer look at chronic viral infections through the lens of viral social interactions and consider how this perspective could increase our understanding of the fundamental biology of these viruses. Following the lead of Leeks et al., (2023) we first define what we mean by an individual in the context of chronic viral infections, before next considering examples of the social interactions taking place during these infections and finally speculating on whether these social interactions are adaptive.

As with other viruses, we can think of viral populations in chronic infections as societies that are made up of individuals, each of which is a physically distinct viral genome (Leeks et al., 2023; Queller & Strassmann, 2009). Whereas acute viral infections only have days or weeks for these societies to develop before they are cleared by host immune responses, chronic infections typically last for years, leading to the potential for complex interactions to develop. In terms of key evolutionary parameters, although acute respiratory viruses, such as influenza virus and SARS‐CoV‐2, have shorter generation times within‐host (i.e. less than half a day), while it takes 2–6 days for HIV‐1, HCV and HBV virions to be released post entry into a cell (Baccam et al., 2006; Harcourt et al., 2020; Perelson et al., 1996; Ribeiro et al., 2012; Schneider et al., 2012; Whalley et al., 2001), within‐host viral loads (i.e. population size) and viral mutation rates are comparable for most acute and chronic viral infections. As such, chronic viral infections provide ideal natural laboratories to study and understand the potential of viral populations to develop societal structures and stable interactions. Many key players populate the within‐host viral population. Relatively less‐studied players within these chronic infections are defective viral genomes, which are generated through several common mechanisms such as point mutations, hypermutations, recombinations, insertions and deletions.

Incomplete genomes with large deletions are the most prominent form of defective genomes, and they almost certainly rely on intact genomes to replicate and to be released into circulation. A recent study found that 37% of the total genetically defective plasma‐derived genomes (26%–51% of all genomes) from both acute/early and chronic HIV‐1‐infected individuals are missing more than 100 bp of their genome (Fisher et al., 2022). A consistent 1%–5% of the HBV genomes in patient sera have been found to have large deletions (Preiss et al., 2008; Soussan et al., 2008). And finally, HCV subgenomes (field‐specific terminology for defective/nonintact genomes) have been observed in circulating plasma and liver tissue from chronically infected patients, with recent studies estimating 19%–26% of HCV patient blood and liver tissue samples containing incomplete genomes (Bernardin et al., 2007; Cheroni et al., 2015; Iwai et al., 2006; Karamichali et al., 2018; Noppornpanth et al., 2007; Ohtsuru et al., 2013; Shimizu et al., 2006; Sugiyama et al., 2009; Yagi et al., 2005).

In addition to circulating defective viral particles, there are also other forms of defective genomes present in these prolonged interactive societies. During its lifecycle, HIV‐1 is reverse‐transcribed into the genome of the immune cell it has infected, creating a provirus. Sometimes these cells enter a long‐lived resting state, forming what is known as the HIV‐1 latent reservoir, and what is the ultimate barrier to a cure for HIV‐1. Over 90% of the HIV‐1 integrated proviral genomes are defective (Bruner et al., 2016), and the proportion of defective genomes with large deletions was found to increase throughout the infection (Pollack et al., 2017). Hepatitis D virus is another example of a defective virus. It hitchhikes on established HBV infections, as its encapsulation requires HBV‐derived envelopes (Urban et al., 2021).

The functions of incomplete genomes are limited by the mRNAs and proteins they encode, but that provides clues for us to understand how they fit into the within‐host societies of these chronic infections. The deletions observed in circulating HIV‐1 defective particles are spread across the entire genome but are more frequent in *env*, which encodes the viral envelope spikes that protrude from the virion envelope, and *pol*, which encodes enzymes that are needed for viral replication (Fisher et al., 2022). Similar patterns have also been found among the proviral defectives with large deletions (Bruner et al., 2016; Ho et al., 2013; Imamichi et al., 2016). In the case of HBV, the ~2.2 kb long defective form with dominating prevalence is reverse transcribed from the major splice variant, which is missing large portions of the polymerase and codes for the core protein (Günther et al., 1997; Sommer et al., 2000). During HBV's life cycle, up to 20 different splice variants could be produced, most of them missing parts of the polymerase and simultaneously the envelope proteins because of overlapping reading frames (Kremsdorf et al., 2021). Finally, the subgenomes described in HCV chronic infections are often the result of in‐frame deletions and mostly lack the envelope proteins as well (Karamichali et al., 2018; Pacini et al., 2009). These defective HCV genomes are usually replication‐competent with intact polymerase coding regions and can be packaged and released into the bloodstream (Pacini et al., 2009; Sugiyama et al., 2009). From the range of proteins coded, or rather not coded, by the incomplete genomes, their social roles appear to revolve around avoiding or enhancing the immune recognition by the host system.

If we are going to use the ideas of sociovirology to frame our thinking around the potential roles of defective genomes, an important starting point is what do we mean by a social trait? In their broad definition of sociality, Leeks et al. (2023) consider a trait to be a social trait if it affects the fitness of another individual. In viral infections, defective genomes have been found to affect the viral population in three ways, interference, immunostimulation and aid in persistence (Vignuzzi & López, 2019). In the context of chronic infections, defective viral genomes are often associated with persistence and in the case of HIV‐1, immunostimulation as well. In one specific example, defective proviral HIV‐1 genomes can produce *gag* and *nef* proteins, which in turn stimulate continuous immune activity in patients on effective combination antiretroviral therapy and who have undetectable virus in their plasma (Ferdin et al., 2018; Imamichi et al., 2016, 2020). Other transcribed products from defective proviruses have also been described as decoys, since they may distract cytotoxic T lymphocytes (CTLs) from eliminating the latent reservoirs by interfering with the CTL killing of cells containing intact HIV‐1 genomes (Kuniholm et al., 2022; Pollack et al., 2017). Intuitively, this is a strategy to attract and exhaust the immune pressure (Kuniholm et al., 2022). However, we speculate that it could also be a mechanism by which the viral population continuously recruits immune cells to be newly infected. In these examples, the defective proviral HIV‐1 genomes could be seen as providing altruistic benefits to the within‐host viral population as a whole, prolonging the infection and maximizing the opportunities for onward transmission.

Circulating HCV genomes with large deletions could be affecting the within‐host viral society's fitness through nonaltruistic social interactions. At first glance, the replication‐competent HCV subgenomes hijacking the envelope packaging produced by intact genomes makes them look like cheaters. However, in contrast to the defective interfering particles found in other viral infections that often appear to be costly (Vignuzzi & López, 2019), HCV subgenomes neither result in lower viral load (Cheroni et al., 2015) nor slow the replication and release of intact HCV particles (Karamichali et al., 2018). Similarly, the presence of defectives with large deletions in HBV infections also appears not to be a burden on the viral population, in that they maintain a stable prevalence of 1%–5% during infection without affecting the intact genomes' viral load (Kremsdorf et al., 2021; Ma et al., 2009; Preiss et al., 2008; Soussan et al., 2008). The maintenance of these subgenomes could be seen as a group trait that is beneficial because it may result in higher rates of gene expression and could also enable the generation of genetic diversity due to relaxed selection, in much the same way that gene duplication is thought to be an important mechanism for generating evolutionary novelty. Moreover, because the subgenomes tend to lack the envelope gene, this will minimize the selective pressure they experience from the immune response.

Given the apparent ubiquitous and often non‐interfering nature of incomplete genomes within chronic viral infections, a natural question to ask is whether these social traits can evolve, or whether they are simply an inevitable consequence of the mistakes made when viruses replicate. For example, if selection is at the level of individual viruses, then we can ask whether individuals with defective genomes have a selective advantage. Hepatitis D virus is a clear example where a defective virus has found a niche, that is HBV infections, where it thrives essentially as a cheat. It is possible that other defective viruses that emerge de novo in each new infection may also be acting as cheaters and derive a selective advantage from this, but we also cannot rule out the null hypothesis that they are simply a common non‐adaptive by‐product of mistakes during replication.

The question becomes more difficult again when we consider potentially altruistic traits that benefit the within‐host population of viruses as a whole, such as by making onward transmission more likely, but are disadvantageous to individuals. Mathematical modelling can often be the first step in evaluating these ideas, particularly when multiple levels of selection are required. For example, it has been shown that the frequency that viruses entering the HIV reservoir could be an evolved trait; most viruses entering the reservoir will have no progeny, but if a small proportion of viruses entering the reservoir are reactivated, and these reactivated viruses have a selective advantage because they are more likely to be successfully transmitted, then the propensity to enter the reservoir can be adaptive at the population level (Lythgoe et al., 2017). However, it is not only necessary to show that a social trait could be positively selected, but that the trait itself is heritable, and for this, there must be genetic variation in the trait. It may be informative to consider the generation of viral decoys through the lens of social interactions, but the production of decoys can only be subject to evolutionary pressures if there is genetic variation in their production among infections.

Chronic viral infections tend to last a long time, making it important for these viruses to outrun or outwit the immune system, but also gives time for social interactions to emerge that could help in this endeavour. Here, we have framed the discussion of social interactions around defective viral genomes. There are many unknowns about their potential roles during chronic viral infection, yet numerous studies have highlighted their underlying importance, although the mechanisms that control the presence and prevalence of these incomplete genomes are as yet unknown. Up to now, defective genomes in chronic viral infections have been relatively understudied, not least because the previous methods used to describe viral genomes have overlooked the defectives within the viral genetic pool (Bruner et al., 2016; Kremsdorf et al., 2021). With increasing awareness of the potential impact, these defectives may have, recent developments in experimental and sequencing techniques have made the description and quantification of defective viral genomes more thorough and accurate (Bruner et al., 2016, 2019; Eriksson et al., 2013; Gaebler et al., 2019; Hiener et al., 2017; Kremsdorf et al., 2021; Reeves et al., 2023; White et al., 2023). With better data, putting the defective genomes in a socio‐evolutionary context will still be a challenging task given the diversity of the forms and functions defective genomes can take, however, the more we understand these interactions, the more mechanisms we will have at hand to advise on novel therapeutics for these chronic viral infections.

## AUTHOR CONTRIBUTIONS

**Lele Zhao:** Conceptualization (equal); writing – original draft (lead); writing – review and editing (equal). **Katrina A. Lythgoe:** Conceptualization (equal); supervision (lead); writing – original draft (supporting); writing – review and editing (equal).

## CONFLICT OF INTEREST STATEMENT

The authors have no conflicts of interest to declare.

### PEER REVIEW

The peer review history for this article is available at https://www.webofscience.com/api/gateway/wos/peer‐review/10.1111/jeb.14244.

## Data Availability

No new data were generated for this manuscript.
